# Patient Perceptions on Receiving Vaccination Services through Community Pharmacies

**DOI:** 10.3390/ijerph19052538

**Published:** 2022-02-22

**Authors:** Anna Kowalczuk, Alexandre Wong, Kevin Chung, Urszula Religioni, Dariusz Świetlik, Katarzyna Plagens-Rotman, Jameason D. Cameron, Agnieszka Neumann-Podczaska, Katarina Fehir Šola, Justyna Kazmierczak, Eliza Blicharska, Regis Vaillancourt, Piotr Merks

**Affiliations:** 1 National Institute of Medicine, 00-725 Warsaw, Poland; a.kowalczuk@nil.gov.pl; 2 Pharmacy Department, Children’s Hospital of Eastern Ontario, Ottawa, ON K1H 8L1, Canada; awong@cheo.on.ca (A.W.); kchung@cheo.on.ca (K.C.); jcameron@cheo.on.ca (J.D.C.); regisvaillancourt@hotmail.com (R.V.); 3 School of Public Health, Centre of Postgraduate Medical Education of Warsaw, 01-826 Warsaw, Poland; 4 Department of Biostatistics and Neural Networks, Medical University of Gdansk, 80-211 Gdansk, Poland; dariusz.swietlik@gumed.edu.pl; 5 Hipolit Cegielski State University of Applied Sciences, 61-485 Gniezno, Poland; plagens.rotman@gmail.com; 6 Chair and Department of Palliative Medicine, Poznan University of Medical Sciences, 61-245 Poznan, Poland; ar-n@wp.pl; 7 Pharmacy of Bjelovar, Petra Preradovića 4, 43000 Bjelovar, Croatia; kfsola@gmail.com; 8 Zdrowit sp. z o.o., Pharmacy Chain, ul. Diamentowa 3, 41-940 Piekary Śląskie, Poland; j.kazmierczak@aptekizdrowit.pl; 9 Department of Analytical Chemistry, Medical University of Lublin, Chodźki 4a, 20-093 Lublin, Poland; elizablicharska@umlub.pl; 10 Faculty of Medicine, Collegium Medicum, Cardinal Stefan Wyszyński University, 01-815 Warszawa, Poland; piotrmerks@googlemail.com

**Keywords:** vaccination, community pharmacy, pharmacist, patient, COVID-19

## Abstract

(1) Introduction: Pharmacists are medical professionals who play an active role in the protection of public health. Since 2021, pharmacists with an appropriate certification have been authorised to administer vaccines against COVID-19. (2) Objective: The objective of this study was to ascertain the perceptions of patients about receiving vaccinations through community pharmacies. (3) Material and methods: This study was conducted in 2021. The research tool was an anonymous questionnaire published on the websites of patient organisations. Ultimately, 1062 patients participated in this study. (4) Results: This study shows that most of the respondents find community pharmacies more accessible than outpatient clinics (85.3%). Sixty-one percent of the respondents stated that getting vaccinated at pharmacies would be less time consuming than at outpatient clinics. Nearly every third respondent (29.5%) declared that they would get vaccinated if they received such a recommendation from a pharmacist. Fifty-six percent of the respondents were of the opinion that the administration of vaccines by pharmacists would relieve the burden on medical staff and the healthcare system. (5) Conclusions: Polish patients participating in the study have a positive attitude towards the implementation of vaccination services in community pharmacies as an effective way of combating infectious diseases.

## 1. Introduction

Pharmacists constitute one of the largest group of medical professionals. According to the Organisation for Economic Cooperation and Development (OECD), there are 77 pharmacists per hundred thousand patients, and 2700 patients per pharmacy in Poland [1,2]. Pharmacists worldwide engage in the protection of public health with respect to health promotion, and by participating actively in the patient treatment process, in cooperation with representatives of other medical professions, particularly doctors and nurses [3,4,5,6,7].

Despite a relatively low level of development in pharmaceutical care in Poland [8] (which is reflected in the small number of services provided by pharmacists), pharmacists express their readiness to expand their services, especially in relation to medicine use reviews, which are considered necessary, even for frail elderly patients [9] and the very elderly with polypharmacy [10], and to administer vaccines [11,12], which, until recently, could only be administered by nurses, following qualification by a doctor [11].

The COVID-19 pandemic has revealed multiple imperfections in the healthcare system, and has posed new challenges, mainly in the fight against infectious diseases [13,14]. A quick response to the current epidemiological situation was required, which was possible due to, for example, close cooperation among the various groups of medical professionals [15]. Since the outbreak of the COVID-19 pandemic, pharmacists worldwide have been actively engaging in patient health protection against this new health threat [14,16,17]. Community pharmacies, fulfilling their role despite the COVID-19 pandemic, have become the most accessible healthcare facilities for patients. For this reason, pharmacists have become the healthcare professionals that patients have often turned to with their health issues [11]. Considering the ease of accessibility of pharmacists (compared to 2.4 physicians per 1000 population) [2], along with the potential of this professional group in relation to the inadequate number of medical staff [18,19,20], regulations were adopted in 2021 in Poland. These regulations allow the expansion of pharmaceutical services to include vaccines against COVID-19, which can be administered by pharmacists after they have acquired relevant certification, following theoretical and practical training.

Given the above, the primary objective of this study was to ascertain the perceptions of patients about receiving vaccinations through community pharmacies, while the secondary objective was to evaluate to what extent these perceptions were conditioned by demographic variables, such as age, gender, education and geographical residence.

The study was approved by the Bioethics Committee of the National Geriatrics, Rheumatology and Rehabilitation Institute in Warsaw (decision no. KBT 3/3/2021—3 March 2021).

## 2. Material and Methods

### 2.1. Data Collection

This study of the perceptions of patients was carried out in 2021. The research tool was an anonymous questionnaire developed for the purposes of this study, consisting of 22 questions on the objective of the study and on personal data. The questions required a single-choice answer from the multiple choices supplied. The questionnaire was constructed based on the literature review and opinions of two experts in research methodology in the field of pharmacy and public health. The questionnaire was distributed online in 2021, with the help of patient organisations and published on their websites (https://mypacjenci.org/en/, http://www.obywatelezz.pl/, http://federacjapp.pl/, https://www.pkpo.pl/ (accessed on 16 November 2021)). Ultimately, 1062 patients took part in the study.

### 2.2. Data Analysis

Statistical analysis was performed with StatSoft. Inc. (Tulsa, OK, USA, 2014) statistic software package, STATISTICA (data analysis software system), version 12.0, www.statsoft.com (accessed on 16 November 2021), and Excel spreadsheet.

Nominal variables were characterised by arithmetical means, standard deviation, minimum and maximum values (range), and a 95% confidence interval. Qualitative variables were presented as size and percentages (share).

A Shapiro–Wilk test was used to assess normality, and a Levene (Brown–Forsythe) test was used to assess the equality of variances.

In order to determine if there was a significant statistical difference between the two groups (independent variables), a Student’s *t*-test (Welch’s *t*-test in the case of inequality on variances) or the Mann–Whitney U test (if conditions for the Student’s *t*-test were not met or for the variables measured at the ordinal scale) were used. The significance of differences between more than two groups was assessed by the F test (ANOVA) or the Kruskal–Wallis test (if conditions for ANOVA were not met). In the case of statistically significant differences between the groups, post hoc tests were used (Tukey’s test for F, and the Dunn’s test for Kruskal–Wallis).

In the case of two connected variables in the model, we used a Student’s *t*-test, or Wilcoxon matched-pairs test (if conditions for the Student’s *t*-test or for the variables measured at the ordinal scale were not met). The significance of differences between more than two groups in the dependent variable model was determined using an analysis of variances with repeated measurements, or a Friedman test (if conditions for the analysis of variances with repeated measurements or for variables measured at the ordinal scale were not met).

For qualitative variables, we used chi-square tests for independence (with Yate’s correction for fewer than 10 cells, evaluation of Cochran conditions and a Fisher’s exact test).

In order to determine the relationship strength and direction between the variables, Pearson and/or Spearman’s correlation coefficients were used. The level of statistical significance was taken at *p* = 0.05.

## 3. Results

### 3.1. Characteristics of the Study Group

The study group distribution was 66.9% women and 30.4% men. The largest group of respondents were 21–40 years of age (49.2%). The largest number of respondents lived in the Mazowieckie region of Central Poland (22.5%). More than 72% of the respondents had higher-level education (Table 1).

### 3.2. Perceptions about Vaccinations in Pharmacies

The patients were asked about how often they visited community pharmacies. More than 42% of the respondents visited a pharmacy 1–3 times a month, and 34.3% several times a year (Table 2). The frequency of visiting a pharmacy increased with the age of the respondents (the correlation coefficient R = −0.07, *p* = 0.0258). A total of 77.3% of the respondents indicated that they lived within 1 km of a pharmacy, and 17.3% within 5 km. Outpatient clinics were located much further away; 60% of the respondents lived within one kilometre of an outpatient clinic.

The respondents reported that they found pharmacies significantly more accessible than outpatient clinics (65.3% strongly agreed and 20% somewhat agreed with this statement). Over 60% of the respondents agreed with the statement that getting vaccinated in a pharmacy would be less time consuming than in a doctor’s office (taking into account the entire process, including travelling to the vaccination point, making an appointment, etc.). The awareness of the fact that administering vaccines in pharmacies takes less time increased with the respondents’ level of education (the correlation coefficient R = −0.11, *p* = 0.0002). The respondents largely agree that vaccinations are the most effective method of fighting infectious diseases, and this awareness increased with the respondents’ level of education (the correlation coefficient R = −0.12, *p* = 0.0001). More than 60% of the respondents agreed (“strongly agree” and “somewhat agree”) that in ageing societies (such as in Poland), a higher vaccination index among elderly people (at higher risk of developing flu-related complications) is more effective at saving lives than treating influenza and its related complications. Again, the respondents’ awareness increased with their level of education (the correlation coefficient R = −0.15, *p* < 0.0001).

Despite this, only 19.8% of the respondents had been vaccinated against influenza in the previous season. Nearly every third respondent (29.5%) indicated that they would get vaccinated against influenza if it were administered by a pharmacist, 17.7% replied “maybe” and 7.4% replied “I don’t know”. A total of 30.3% of the respondents (30.3%) declared that they were going to get vaccinated against influenza in the next flu season (2021/2022), while 26.6% were undecided. A total of 42.6% of the respondents were going to get vaccinated against COVID-19 (of which 15.3% were undecided).

Most of the respondents (60.0%) agreed with the statement that vaccines administered by trained pharmacists would relieve the burden on doctors and nurses, and this awareness increased with the level of education (the correlation coefficient R = −0.11, *p* = 0.0006). A similar percentage of the respondents (56.2%) agreed with the statement that easier access to vaccinations against influenza would have a positive impact on the healthcare system (e.g., by reducing the number of hospitalisations and visits to the doctor for treatment of influenza and its resulting complications). Again, it was observed that the respondents with a higher level of education agreed with this statement significantly more often (the correlation coefficient R = −0.13, *p* < 0.0001).

The last question concerned the respondents’ experiences with their last vaccination administered at an outpatient clinic, before it was possible to receive this service in a pharmacy. Only 29.9% of the respondents strongly agreed with the statement that their last vaccination was a positive experience. Every fourth respondent (23.2%) did not rate this service positively.

## 4. Discussion

Our study indicates that over 76% of the respondents in the study visit a community pharmacy at least several times a year. The majority of the respondents live within a short distance of a pharmacy, which favours health promotion activities in this area. This is of particular importance for elderly people, who live closer to pharmacies than younger people, and to people with a lower level of education, who, in our study, were less aware of the role of vaccinations in preventing infectious diseases.

The percentage of the respondents expressing their readiness for vaccinations administered by pharmacists is of key importance. Though 30% of the respondents expressed this readiness, 25% of the patients hesitated. If pharmacists promote vaccinations and provide relevant information, this group could become the next large group to receive vaccinations in community pharmacies.

The perceptions of patients about their last experience receiving a vaccination is significant. Nearly 30% of the respondents agreed that getting vaccinated was a positive experience, which can have an influence on their decisions regarding future vaccinations.

Vaccinations are the most effective method for preventing infectious diseases [21] that have serious health consequences. This is particularly important in ageing populations, and when taking into account the inadequate number of medical staff. This situation is extremely important in light of the COVID-19 pandemic and the availability of vaccinations against coronavirus that can largely reduce the spread of the pandemic and prevent the negative consequences of this disease [22]. Vaccinations against COVID-19 are recommended, by the World Health Organisation (WHO), as the method for preventing the pandemic [23]. Despite the increasing accessibility of vaccinations and the numerous information campaigns for patients, the proportion of people fully vaccinated against COVID-19 is 52.61% (as of November 2021). This figure is considerably lower than the European Union average (64.94%) [24]. Evidence in the literature indicates that access to information on the pandemic, the consequences of this disease, and prevention possibilities have a large impact on decisions regarding vaccinations, thereby increasing the vaccination coverage of the population [25,26].

Considering the wide access to pharmacies and pharmacists, and the high level of competency of this medical group, community pharmacies can contribute to a substantial increase in the vaccination coverage level [27].

Protective vaccinations have already been implemented in multiple European countries (including Great Britain, Ireland, France, Switzerland, Denmark, Greece and Portugal) [28]. The data provided by WHO indicate that vaccinations against influenza are available in about 40% of pharmacies in Europe, half of which also provide other vaccinations [29]. It is worth mentioning Great Britain, where vaccinations against influenza were implemented in community pharmacies in 2002, and the vaccination coverage level was 75%. These data emphasise the considerable impact that pharmacists, providing vaccinations, can have on relieving the burden on the healthcare system [30]. Expanding pharmaceutical services to include vaccinations is beneficial for patients and healthcare systems [31]. Resistance to infectious diseases prevents multiple health complications, minimises the risk of hospitalisation, and reduces the costs of the healthcare system.

Increasing vaccination coverage levels requires the engagement of many medical professionals [32]. Pharmacists can play a huge role in achieving this objective, as they can increase the availability of vaccinations to the population, which is one of the key conditions that leads to higher vaccination levels [33,34]. Previous studies show that the implementation of this service to community pharmacies can increase the percentage of vaccinated people, compared to the traditional system of administering vaccines in outpatient clinics [33]. The lack of accessibility of community pharmacies with long opening hours, and the lack of necessity to make appointments, largely favours the patients and can positively influence their decisions to get vaccinated [35].

In order to prepare pharmacists for administering vaccines, it is essential to enable them to obtain the required theoretical and practical qualifications. Pharmacies need to have the relevant equipment to meet the organisational requirements and ensure patient privacy. Another key factor is connected with the financing of vaccinations (e.g., by public payers). Information campaigns, directed to the largest target group possible, play an important role in promoting vaccinations in pharmacies [7,36].

## 5. Limitations

Our study has several limitations. First of all, we had no influence on the distribution of demographic characteristics of the people participating in the study. They were certainly people who use the internet. Thus, we do not know the opinions of people who do not use a computer/telephone with internet access on a daily basis, or do not visit the portals indicated in this study, etc. Therefore, the obtained results and findings cannot be generalised to the entire Polish population. The current study design was the only way to meet the stated study objective. There are certainly other designs that can be used to obtain a more nationally representative sample. Moreover, our results could be influenced by the time of implementation of the study, which coincided with the beginning of obtaining authorisation for vaccinations by pharmacists.

## 6. Conclusions

The respondents in our study indicate that they regularly visit community pharmacies. The vast majority of them declare that they would get vaccinated if vaccines were available from pharmacists. Vaccinations against COVID-19 in a number of community pharmacies are an effective tool in the fight against the coronavirus pandemic. Authorising pharmacists to administer vaccines in Poland has revealed the potential of this professional group for the protection of public health. Given the above, further expansion of pharmaceutical services in Poland seems to be necessary in order to provide the most comprehensive patient care possible.

## Figures and Tables

**Table 1:** Characteristics of the study group.

Characteristic	Study Group (*n* = 1062)
Age	
0–20 years	45 (4.2%)
21–40 years	522 (49.2%)
41–60 years	336 (31.6%)
61–80 years	155 (14.6%)
81 years and older	4 (0.4%)
Gender	
Male	323 (30.4%)
Female	710 (66.9%)
Prefer not to answer	24 (2.3%)
Other	5 (0.4%)
Region	
Mazowieckie	239 (22.5%)
Pomorskie	68 (6.4%)
Lubuskie	21 (2.0%)
Małopolskie	70 (6.6%)
Podkarpackie	41 (3.9%)
Dolnośląskie	59 (5.6%)
Kujawsko-pomorskie	88 (8.3%)
Wielkopolskie	79 (7.4%)
Lubelskie	50 (4.7%)
Podlaskie	32 (3.0%)
Warmińsko-mazurskie	42 (4.0%)
Śląskie	130 (12.2%)
Łódzkie	89 (8.4%)
Zachodnio-pomorskie	34 (3.2%)
Świętokrzyskie	5 (0.5%)
Opolskie	15 (1.4%)
Education	
primary	9 (0.8%)
lower-secondary	6 (0.6%)
basic vocational	22 (2.1%)
secondary	258 (24.3%)
higher	767 (72.2%)

**Table 2:** Selected issues regarding the perceptions of patients about vaccinations.

How Often Do You Visit a Pharmacy?	Study Group (*n* = 1062)
Very often (at least twice a week)	50 (4.7%)
Often (once a week)	66 (6.2%)
Sometimes (1–3 times a week)	454 (42.7%)
Seldom (a few times a year)	364 (34.3%)
Very seldom (once a year or less)	109 (10.3%)
Never	19 (1.8%)
Do you agree with the statement that pharmacies are more accessible for patients than outpatient clinics?	Study group (*n* = 1062)
Strongly agree	693 (65.3%)
Somewhat agree	212 (20.0%)
Neither agree nor disagree	105 (9.9%)
Somewhat disagree	28 (2.6%)
Strongly disagree	24 (2.3%)
Do you agree with the statement that getting vaccinated in a pharmacy would be less time consuming than in a doctor’s office?	Study group (*n* = 1062)
Strongly agree	398 (37.5%)
Somewhat agree	249 (23.4%)
Neither agree nor disagree	162 (15.3%)
Somewhat disagree	70 (6.6%)
Strongly disagree	183 (17.2%)
Do you agree that vaccinations are the most effective method for preventing infectious diseases?	Study group (*n* = 1062)
Strongly agree	460 (43.3%)
Somewhat agree	209 (19.7%)
Neither agree nor disagree	74 (7.0%)
Somewhat disagree	99 (9.3%)
Strongly disagree	220 (20.7%)

## Data Availability

All data are available from the corresponding author.
